# Identification Framework of Contaminant Spill in Rivers Using Machine Learning with Breakthrough Curve Analysis

**DOI:** 10.3390/ijerph18031023

**Published:** 2021-01-24

**Authors:** Siyoon Kwon, Hyoseob Noh, Il Won Seo, Sung Hyun Jung, Donghae Baek

**Affiliations:** Department of Civil and Environmental Engineering, Seoul National University, 1 Gwanak-ro, Gwanak-gu, Seoul 08826, Korea; ksy92@snu.ac.kr (S.K.); hyoddubi1@snu.ac.kr (H.N.); jsungh@snu.ac.kr (S.H.J.); wes1204@snu.ac.kr (D.B.)

**Keywords:** contaminant source identification, transient storage zone model, breakthrough curve analysis, ensemble decision tree model, recursive feature elimination cross-validation, tracer test

## Abstract

To minimize the damage from contaminant accidents in rivers, early identification of the contaminant source is crucial. Thus, in this study, a framework combining Machine Learning (ML) and the Transient Storage zone Model (TSM) was developed to predict the spill location and mass of a contaminant source. The TSM model was employed to simulate non-Fickian Breakthrough Curves (BTCs), which entails relevant information of the contaminant source. Then, the ML models were used to identify the BTC features, characterized by 21 variables, to predict the spill location and mass. The proposed framework was applied to the Gam Creek, South Korea, in which two tracer tests were conducted. In this study, six ML methods were applied for the prediction of spill location and mass, while the most relevant BTC features were selected by Recursive Feature Elimination Cross-Validation (RFECV). Model applications to field data showed that the ensemble Decision tree models, Random Forest (RF) and Xgboost (XGB), were the most efficient and feasible in predicting the contaminant source.

## 1. Introduction

When accidental spills of contaminant occur in natural rivers, a rapid response is necessary to minimize the damage to both aquatic life and humans who depend on the river as a water resource. Contaminant accidents in rivers are risky and urgent problems that occur frequently, mainly by transportation accidents or industrial waste [1,2,3]. In this respect, quick identification of the contaminant source plays a significant role in protecting river systems and environmental forensic by providing information of the contaminant source, such as spill location, spill mass, and release history. However, inverse tracking of the contaminant source is a problem, due to the lack of observed data and complexity of the mixing processes in a natural river. In order to overcome this limitation, a number of methods for the identification of contaminant sources have been suggested, mainly in the groundwater system; these use various techniques, such as optimization, geostatistical simulations, analytical solutions, and data-driven models [4,5,6,7,8,9,10,11,12,13,14,15]. Although contaminant source identification problems in both rivers and groundwater have a similar purpose, applying the methods developed for groundwater to rivers is challenging, due to the difference in flow and mixing characteristics between groundwater and rivers. Specifically, a quick response is more crucial in rivers than in groundwater, since the contaminants are transported more rapidly in rivers than in groundwater.

Among many inverse tracking methods used in the groundwater system, the optimization method was frequently used in river systems, which iterates the calculations based on the advection–diffusion process to reach the global solution of contaminant source as an ill-posed problem. Parolin et al. [16] carried out a hybrid heuristic algorithm, which included the Luus–Jaakola method (LJ), particle collision algorithm (PCA), ant colony optimization (ACO), and golden section method (GS), to identify the spill location and intensity of contaminant source in an estuary. Zhang and Xin [17] used the basic Genetic Algorithm (GA) to identify the spill location and spill mass of contaminant sources in a small straight river. However, these optimization approaches have limitations of high uncertainties in their deterministic processes and the data used in the optimization [18]. Thus, stochastic methods based on Bayesian inference were suggested to overcome the disadvantage of deterministic optimization. Yang et al. [3] combined the Differential Evolution Algorithm (DEA) and Metropolis–Hastings–Markov Chain Monte Carlo (MH–MCMC) to enhance the optimization process with noise immunity. Nevertheless, computational loads of these methods to predict the contaminant source were too expensive to apply in real-time, and high inverse uncertainty occurred according to the objective function in the optimization procedure [18].

Cheng and Jia [19] suggested a backward location probability density function (BL–PDF) to identify the spill location. They evaluated the proposed method regarding noise, and validated the model with the data from the real dye tracer test performed in the natural river, which is a significant process to test field applicability. Ghane et al. [20] also applied the backward probability method further to predict the release time, while Boano et al. [21] employed a geostatistical method to recover the release history under the assumption that the spill location was known. In order to improve the performance of the stochastic model, the Ensemble Kalman filter was coupled with backward location probability [2]. In terms of the uncertainty of the identification results, these stochastic-based methods were proven to be more applicable to the contaminant source identification problems than deterministic-based methods [2,3,20,22].

Despite the valid performance of these stochastic methods, reflecting the complex mixing characteristics in inverse tracking models is very intricate, because the advection–diffusion process contains many problems of spatial and temporal scale. For this reason, data-driven approaches using contaminant spill scenarios to identify the location of the contaminant source were recently presented. The data-driven model extracts the scenario that best matches the observed data, which is obtained downstream of the spill location. This approach has the advantage that the scenarios would include the river mixing mechanisms via model parameters, and the spatial and temporal scales would be explicitly calculated. Telci and Aral [23] simulated contaminant spill scenarios in the Altamaha River, USA, and they developed a sequential feature selection algorithm using the scenarios, which sequentially eliminates potential spill locations in the scenarios. Kim et al. [24] and Lee et al. [25] employed the Random Forest (RF) method to build a spill location predictor, using the same contaminant spill scenarios used by Telci and Aral [23]. Compared to the other methods, the data-driven models require a low computational load for prediction, even though the training process requires a large dataset [26]. In this regard, the data-driven models are more feasible for the real-time prediction of a contaminant spill, facilitating a quick response to river spill accidents. As RF was used above, Machine Learning (ML) techniques have been widely utilized in data-driven models to investigate the complex functional relations in water resources [27,28,29,30,31,32,33].

A significant factor in determining the performance of the contaminant identification model using a data-driven model is the reality of the contaminant scenarios. However, the previous studies [23,24,25] have a disadvantage, since the Storm Water Management Model (SWMM), which assumed the Continuous flow Stirred Tank Reactors (CSTR), was used as a contaminant transport model [23,34]. Such a simplified model would be incapable of accurate simulation of complex hydrodynamics and contaminant transport in rivers.

This study presents an enhanced framework for the identification of a contaminant source in rivers. The first objective of the proposed framework was to generate realistic contaminant spill scenarios. For this objective, the Transient Storage zone Model (TSM) was used as a contaminant transport model to generate the contaminant spill scenarios. This model has been successfully used to reproduce the breakthrough curve (BTC), which is a time-concentration curve of the contaminant that represents the mixing processes with advection, shear dispersion, and storage effect in the river [35,36,37,38,39]. The second objective was to develop Machine Learning (ML) models for the identification of both spill location and mass of the contaminant source in rivers. The contaminant spill scenarios calculated by TSM were used as training and validation dataset. In this procedure, 21 features extracted from the BTCs of spill scenarios were used to predict the contaminant source by the six ML models. The optimal BTC features of both spill location and spill mass predictions were selected by Recursive Feature Elimination Cross-Validation (RFECV), which selected features recursively according to the feature importance of the ML model. Finally, the proposed models were applied to the field tracer data obtained in the river in order to ascertain the field applicability.

## 2. Methodology

**Figure 1:**
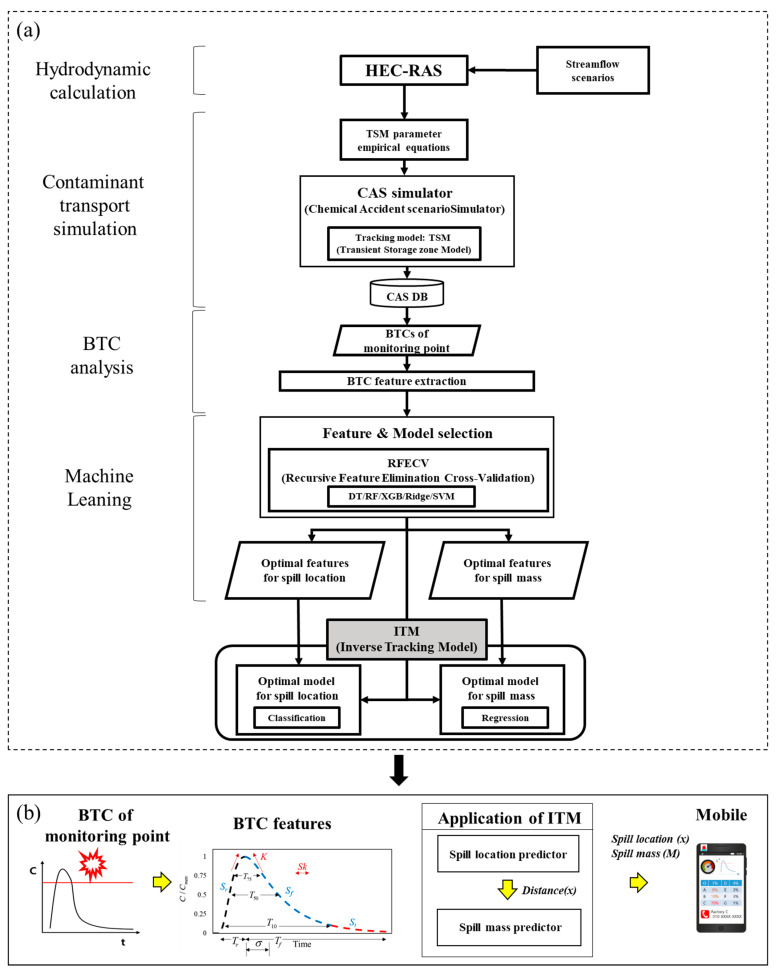
Schematic of the Inverse Tracking Model (ITM) framework: (**a**) development, and (**b**) application; HEC-RAS (Hydrologic Engineering Center’s River Analysis System) and TSM (Transient Storage Zone model) are hydrodynamic and contaminant transport model; CAS is Chemical Accident scenario Simulator; DB is Data Base; BTC is BreakThough Curve; DT is Decision Tree; RF is Random Forest; XGB is Xgboost; Ridge is Ridge regression model; SVM is Support Vector Machine.

The Figure 1a shows a flowchart of the development of the proposed framework of the Inverse Tracking Model (ITM) to identify the spill location and mass of a contaminant source. The framework consists of four steps: hydrodynamic calculation, contaminant transport simulation, BTC analysis, and ML modeling.

Section 2.1 describes the first and second steps, in which the Contaminant Accident Scenarios Data-Base (CAS DB) is developed by numerical models of river hydrodynamics (HEC-RAS) and contaminant transport (TSM). Section 2.2 explains the third step, which includes the BTC analysis. This step features the BTCs of monitoring points to build the ITM. The BTC features serve as training and validation dataset of the ITM, instead of the BTC itself. Section 2.3 describes the last step of the ML process in detail. The ITM uses the classification and regression model of ML to build models that predict the spill location and mass of the contaminant source, respectively. In this process, the optimal ML model and BTC features are selected through RFECV.

Figure 1b indicates the application process of the proposed ITM. When the BTC is detected from the sensor at the downstream of the spill location, the BTCs serve as the input data of the ITM. Then, this observed BTC is reduced into BTC features, which are substantial input variables of the ITM. Upon receiving the input data of the BTC features, the spill location is first predicted, and then the spill mass is predicted by adding the spilled distance to the BTC features through the predicted spill location.

### 2.1. Contaminant Accident Scenarios (CAS)

#### 2.1.1. Transient Storage Model (TSM)

In most of the natural river, various types of transient storage zones, called dead zones or stagnant zones, exist along and across the stream, of which the effects cannot be modeled by the conventional one-dimensional advection–dispersion equation (1D ADE) [40]. In contrast to the main free-flowing water zone where the advection and dispersion mechanisms are dominant, the storage zone that is created by various channel irregularities, such as pools, side pockets, vegetation, hydraulic structures, and hyporheic zone, can be defined as an area where the flow is stagnant or recirculated. With respect to contaminant transport, the storage zone effect induces the shape of the BTC to present a steep slope in the rising limb, and a long tail in the falling limb. This skewness of the BTC arises due to transient trapping of contaminants in the storage zone. Since each stream has its own storage zone characteristic, the observed BTC represents the mixing properties of the stream. Therefore, the TSM generates a more realistic BTC for the non-Fickian transport processes than the 1D ADE-based model, by reflecting the storage zone effect [35,41,42].

The TSM consists of two equations: the main free-flowing water zone equation, and the storage zone equation. The equations are modified version of ADE to describe the storage effect by conceptually dividing the area into the main flow zone area ($A_{F}$), and the storage zone area ($A_{S}$). It also exhibits a mass exchange rate (α), which is a first-order mass transfer between the main flow zone and the storage zone. Based on the assumption of steady uniform flow, conservative solute, and completely mixed storage zone, the equations are given as [35,37]:

(1) $$\frac{\partial C_{F}}{\partial t}=-\frac{Q}{A_{F}}\frac{\partial C_{F}}{\partial x}+\frac{1}{A_{F}}\frac{\partial }{\partial x}(A_{F}K_{F}\frac{\partial C_{F}}{\partial x})+\frac{q_{L}}{A_{F}}(C_{L}-C_{F})+\alpha (C_{S}-C_{F})$$

(2) $$\frac{dC_{s}}{dt}=\alpha \frac{A_{F}}{A_{S}}(C_{F}-C_{S})$$

where $C_{F}$, $C_{s}$, $C_{L}$ are the concentration of the main flow zone, storage zone, and lateral flow concentration, respectively [kg/m^3^]; *t* is time [*s*]; *x* is distance [*m*]; Q is the volumetric discharge of the stream [m^3^/s]; $K_{F}$ is the longitudinal dispersion coefficient of the main flow zone [m^2^/s]; $q_{L}$ is lateral inflow rate [m^2^/s]; $A_{F}$ and $A_{S}$ are the cross-sectional area of the main flow zone and storage zone, respectively [m^2^]; and α is the exchange rate of the storage zone [1/s].

In real river systems, $K_{F}$, $A_{F}$, $A_{S}$, and α in TSM equations are unmeasurable parameters, because the storage zones in each stream vary significantly. Thus, in most studies, the exact values of these four parameters were estimated using the optimization method from field tracer data [43,44,45]. With respect to spill scenarios, Rivord et al. [46] employed One-dimensional Transport with Inflow and Storage (OTIS) [37] to model contaminant spills in the Truckee River. They considered only the dispersion process using dispersion coefficients ($K_{F}$) estimated by empirical equations with streamflow (*Q*), reach slope (*S*), and cross-sectional area (*A*). Although they estimated $K_{F}$ under various streamflow conditions using the empirical equation, when storage effects were not considered, their results showed relatively large errors.

To overcome this limitation, empirical equations for TSM parameters have recently been derived from a meta-analysis of river mixing tracer tests [39,47]. From these equations, the TSM parameters can be estimated using easily measurable hydraulic and geometry variables. Thus, in this study, Principal Component Regression (PCR)-based empirical equations for TSM parameters were used to estimate TSM parameters. Equation (3) gives the equation, while Table 1 gives the derived power [39]:

(3) $$\left(\frac{K_{F}}{hU_{*}},\frac{A_{F}}{Wh},\frac{A_{S}}{Wh},\frac{\alpha }{U_{*}/h}\right)=\exp(a){\left(\frac{U}{U_{*}}\right)}^{b}{\left(\frac{W}{h}\right)}^{c}{\left(S_{n}\right)}^{d}$$

where *W* is the channel width [*m*]; *h* is the mean flow depth [m]; U is the mean flow velocity [m/s]; *Sn* is the channel sinuosity; and $U_{*}$ is the shear velocity, which is estimated from the following equation: $U_{*}=\sqrt{ghS_{0}}$, where *g* is the gravitational acceleration [m/s^2^] and $S_{0}$ is the mean bottom slope.

In this study, MATLAB-based TSM code was employed [48]. This model used the finite difference method and the Crank–Nicolson method, similar to the OTIS by Runkel [37].

#### 2.1.2. CAS Simulation

In order to generate scenarios under various streamflow and spill conditions, it is necessary to simulate a wide range of contaminant spill and flow cases in the range that may occur in the river system. Accordingly, the streamflow scenarios were generated by estimated streamflow distribution from the historical data of the study site. In this study, the 1-D Hydrologic Engineering Center-River Analysis System (HEC-RAS) (US Army Corps of Hydraulic Engineers, Washington, DC, USA), was used to calculate hydraulic and geometric information from the streamflow scenarios for the input variables of TSM empirical equations. The HEC-RAS calculates 1-D unsteady flow by solving the Saint Venant equations according to input data of initial flow rate, lateral flow, topographic data of cross-sectional shapes, and roughness coefficient [49]. In this framework, the flow regime was assumed to be steady uniform flow within sub-reach, and steady nonuniform flow considering lateral inflow from a tributary.

The contaminant spill conditions were assumed to be an instantaneous injection with conservative contaminants that do not decay. The spill mass was generated randomly from a uniform distribution. In particular, the 1D ADE analytical solution of the instantaneous injection was applied to the upstream boundary concentration [40]. From this approach, spill mass can serve as an input variable of TSM simulation. Due to the initial condition given by $C_{F}(0,t)=\infty$, the upstream boundary condition was assumed to be that shortly after the contaminant spill, the storage zone effect does not exist. Thus, the upstream boundary concentration profile at 10 m away from the spill location as $C_{F}(10,t)$ was used for the initial boundary condition:

(4) $$C_{F}(x,t)=\frac{M}{\sqrt{4\pi K_{F}t}}\exp\left[\frac{-{\left(x-U_{F}t\right)}^{2}}{4K_{F}t}\right]$$

where *M* is the spill mass [kg].

In order to build the ITM, a large number of contaminant spill scenarios were required. Thus, we developed a CAS simulator using the Parallel for Loops (parfor) in MATLAB’s Parallel Computing Toolbox, which provides more efficient simulation using shared-memory parallelization of the calculations on multicore-processor CPUs. In CAS, a large amount of scenario cases were simulated, according to spill locations and streamflow scenarios.

### 2.2. Breakthrough Curve (BTC) Analysis

**Figure 2:**
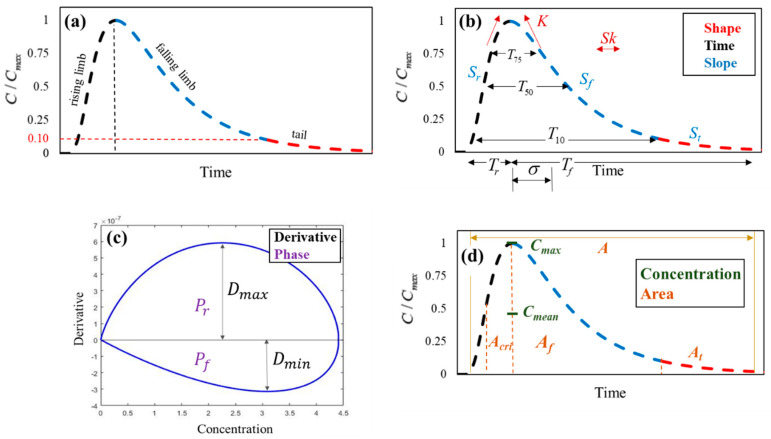
(**a**) Typical BTC and BTC features for (**b**) shape, time, and slope, (**c**) derivative and phase, and (**d**) concentration and area.

Figure 2a shows a hypothetical breakthrough curve (BTC) of the in situ river monitoring sensor from an instantaneous injection [50,51]. The BTC, which is a temporal distribution of contaminant concentration obtained from the monitoring sensor, consists of a rising limb, falling limb, and tail, as depicted in Figure 2a. In this study, the tail in the falling limb is defined as the portion of which the concentration is below the value of 0.1 maximum concentration of BTC as shown in Figure 2a. Although the ideal shape of BTC based on ADE is a bell shape, the actual shape of BTC in rivers is asymmetrical due to the complexity of the flow mechanism and the river morphology, including the storage effect in natural rivers. Furthermore, the BTC implies hydraulic and geometry characteristics due to the passive behavior of contaminants in the stream when the contaminants reach the in situ sensor. For this reason, the BTC can be used as relevant information to track contaminant source inversely. Therefore, in this study, the various features were extracted from the BTC, and those features served as input variables of the ML modeling for the development of the ITM, as shown in Figure 1. This approach enhances the accuracy of the ML model by removing the irrelevant information of the BTC, which also makes the models more efficient by reducing the dimension of input variables. Consequently, in this study, the BTC was characterized by 21 features, as shown in Figure 2 and Table 2.

The features are categorized into shape, concentration, slope, time, integral, derivative, and phase features, as shown in Figure 2b–d. First, the shape features, which are widely used for analyzing the BTC [23,24,52,53], were calculated from the third and fourth temporal moments that indicate the asymmetry and peak of the BTC. The equations to calculate the features and the temporal moment are as follows:

(5) $$m_{k}=\int_{0}^{\infty }t^{k}C(x,t)dt$$

(6) $$Sk=\frac{m_{3}}{m_{2}{}^{3/2}}$$

(7) $$K=\frac{m_{4}}{m_{2}{}^{2}}-3$$

(8) $$\sigma =\sqrt{m_{2}}$$

where *m* is the temporal moment and *k* is the degree of the moment; the other notations are given in Table 2. The temporal moments were estimated using the trapezoid rule [23].

Secondly, the slope features were applied to the segments of BTC, of rising limb, falling limb, and tail. The slope of the rising and falling limb was calculated by dividing the maximum concentration by the time variation of each part. These features indicate how quickly the contaminant increases and decreases. Thus, if advection is more dominant than dispersion, the peak concentration is increased, and the retention time is decreased, which is equivalent to the slope being increased. In particular, the magnitude of the storage zone effect from the contaminant retention is featured as the power-law shape, described in previous studies [54,55]. For this reason, the tail slope was calculated by the power of the equation from the power-law regression.

Next, the time features include the standard deviation and duration of concentration. The Standard deviation quantifies the variance of BTC, which is calculated from the second moment, as shown in Equation (8). Moreover, durations refer to the time needed for the concentration to reach a specific percentage of maximum concentration, and the width of the rising and falling limb. The duration of a specific concentration indicates for how long the concentration stays above the reference concentration. The integral features are the area of each part of the BTC. In addition, we suggest a critical area where the maximum concentration passes from half the maximum concentration in the rising limb, which is defined as the most damaging area.

On the other hand, the derivative and phase features were estimated in phase space, which generates the novel features from the time dependence of the scalar quantity [56]. The phase space was defined so that the concentration and the first derivative are coordinated, as shown in Figure 2c [57]. In this space, the absorption and desorption processes in chemical sensors were characterized. Therefore, we employed the maximum derivative value and the area of positive value in the phase space as features of the rising limb; moreover, we selected the minimum derivative value and the area of negative value in the phase space as features of the falling limb. The phase features can be defined as Equation (9):

(9) $$P=\int_{C(t_{i})}^{C(t_{i+1})}DdC$$

where *C*(*t_i+_*_1_) and *C*(*t_i_*) are the concentration at time *t_i+_*_1_ and *t_i_*.

### 2.3. Machine Learning (ML) Modeling

**Figure 3:**
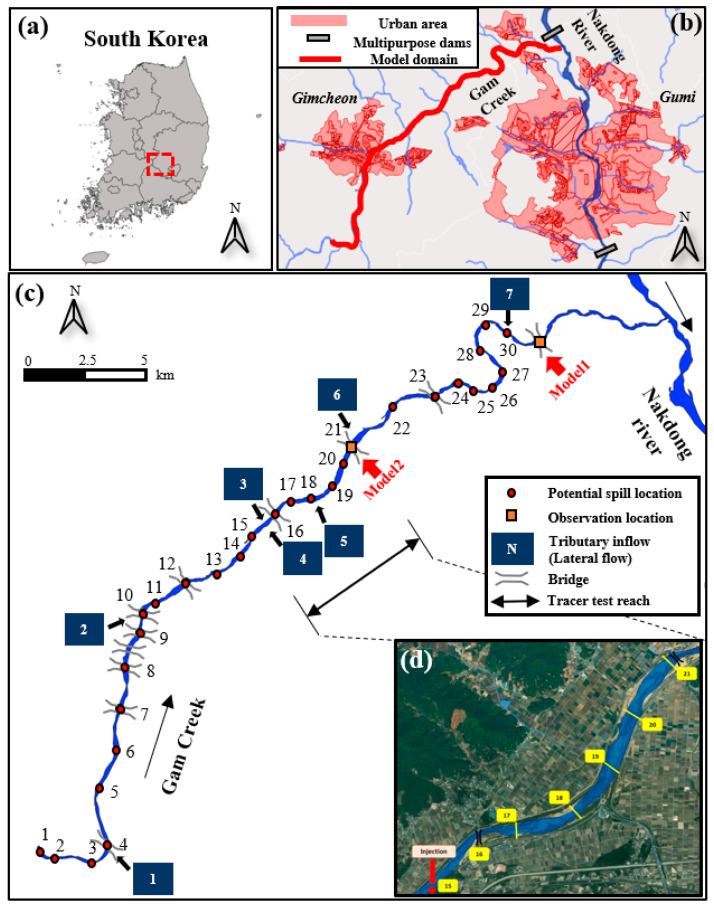
Site map showing (**a**) the location of the Gam creek region in South Korea; (**b**) the model domain and the location of urban areas that would potentially be subject to significant damage from the spilled contaminants near the region; (**c**) the potential spill locations and monitoring points developing ITM in the Gam Creek region; (**d**) the tracer test reach.

In this framework, we focused on the optimal BTC features and ML models to predict the spill location and spill mass. We conducted six ML models, which consisted of three decision tree-based models: Decision tree (DT), Random Forest (RF), and XGBoost (XGB); two linear models: Ridge and linear Support Vector Machine; and a nonlinear SVM using the Radial Base Function (RBF) kernel. For the prediction of both spill location and mass, predictors were separately developed by classifiers and regressors of the ML models. First, the spill location predictor was developed by using a classification model, because the spill location is labeled as discrete integers, as shown in Figure 3c, which present the potential spill location. In contrast, the spill mass is represented by continuous values as quantities. Thus, the spill mass predictor was developed by using the regression algorithms. Additionally, although both predictors were trained by using the same BTCs at the monitoring point, the optimal BTC features to predict two target variables were investigated separately. All of these models were implemented as both regressors and classifiers using the Scikit-learn and XGBoost libraries in Python 3.7 (Python Software Foundation, Beaverton, OR, USA).

#### 2.3.1. DT-Based Models

The DT is a non-parametric model, and is used as both a classification model and a regression model [58]. This model divides the space of the input variable into multiple hierarchies according to the value of the output variable based on the tree structure. The prediction is performed by taking the mode or average of the output variables through the hierarchy. In the training process of this model, the splitting variables and the split nodes are determined by the Gini index, as given in Equation (10):

(10) $$Gini=\sum_{k=1}^{K}{\hat{p}}_{mk}(1-{\hat{p}}_{mk})$$

where *K* represents the number of classes in the label and ${\hat{p}}_{mk}$ is the proportion of the *k*th class in the node *m*.

This model has the following advantages: (a) Ease of investigating the process of prediction; (b) Insensitivity to noise and truncated data; and (c) High efficiency—it takes a short time to build the model and gives a short-term prediction. Due to these reasons, DT is suitable to be applied to a chemical accident response system that requires rapid forecasting.

In this study, the advanced DT-based algorithms, such as RF and XGB, were also developed to overcome the disadvantage of DT having a high variance of prediction. RF consists of ensemble learning by combining a large collection of DTs, and obtains the results by averaging or voting [59]. Specifically, each DT predictor of RF is developed from a random selection of samples and variables. This process is based on the Bagging (abbreviation for bootstrap aggregation) method proposed by Breiman [60]. It generates the sample by a bootstrap sampling, which samples randomly with replacement. Thus, the Bagging method with randomization reduces the variance of RF by reducing the correlation between the trees. With respect to regression, this model is performed by averaging the predictions of each DT. Otherwise, the classification model is performed by obtaining the majority class vote from the results of each DT.

On the other hand, Chen and Guestrin [61] recently suggested XGB to improve the performance of DT. This model is also an ensemble learning method of DT, and appeared as the top model in various machine learning comparison studies [62,63]. The difference from RF is that XGB is based on the gradient boosting method. In the gradient boosting method, each DT of XGB is developed at an iteration to reduce the error. Thus, XGB integrated multiple DTs into one strong predictor having sequential structure with randomization. In comparison with the conventional gradient boosting method, XGB is the stepwise forward additive model by including a regularization term in the objective function. In addition, it automatically utilizes the multicore and distributed settings for an efficient training process [64,65].

In the XGB, additive functions to predict the output voted or averaged by a collection *F* of *k* trees can be written as:

(11) $$\hat{y}=\sum_{k}^{K}f_{k}\left(x_{i}\right),f_{k}\in F$$

The objective function with loss function and regularization term is used to correct the previous DT through the iteration for optimization, which is given by:

(12) $$L\left(\varphi \right)=\sum_{i}l(\hat{y},y_{i})+\sum_{k}\Omega \left(f_{k}\right)$$

where *l* is a loss function that measures the error between the prediction value (${\hat{y}}_{i}$) and the target value ($y_{i}$), and Ω(f) is a regularization term that describes the complexity of DT $f_{k}$, which is defined as:

(13) $$\Omega (f)=\gamma T+\frac{1}{2}\lambda {\left\|w\right\|}^{2}$$

Due to the complexity of learning all DT parameters at once, the prediction value (${\hat{y}}_{i}$) is given from additive training, which adjusts the current state for the iteration t from the previous iteration *t −* 1:

(14) $${\hat{y}}_{i}{}^{\left(t\right)}=\sum_{k=1}f_{k}(x_{i})={\hat{y}}_{i}{}^{\left(t-1\right)}+f_{t}(x_{i})$$

where γ is the complexity of tree leaf in the DT, T is the number of leaves in the DT, λ is the scale parameter, and w is the scores vector of leaves in the DT. By substituting Equation (14) into Equation (12), the objective function is described as Equation (15). Then, the objective function can be simplified to Equation (16), by taking the second-order Taylor expansion:

(15) $$L^{\left(t\right)}=\sum_{i=1}^{n}l\left(y_{i},{\hat{y}}_{i}{}^{\left(t-1\right)}+f_{t}(x_{i})\right)+\Omega (f_{t})$$

(16) $$L^{\left(t\right)}≅\sum_{i=1}^{n}\left[g_{i}f_{t}(x_{i})+\frac{1}{2}h_{i}f_{t}^{2}(x_{i})\right]+\Omega (f_{t})$$

where $g_{i}=\partial_{{\hat{y}}^{\left(t-1\right)}}l\left(y_{i},{\hat{y}}^{\left(t-1\right)}\right)$ and $h_{i}=\partial_{{\hat{y}}^{\left(t-1\right)}}l\left(y_{i},{\hat{y}}^{\left(t-1\right)}\right)$.

#### 2.3.2. SVM and Ridge Regression

The SVM is a widely used algorithm for both classification and regression. The SVM uses a hyperplane determined by support vectors to classify labeled datasets, which determines the decision boundary of all classes [66]. An optimal hyperplane is a classification plane obtained from the maximum classification margin. It can be obtained from the decision function of SVM in Equation (17). The margin is $\frac{2}{\left\|w\right\|}$, which can be maximized by minimizing the ${\left\|w\right\|}^{2}$. Thus, the optimization problem can be transformed into a dual problem through the Lagrange optimization method (Equation (18)):

(17) f(x)=w⋅x+b

(18) $$L=\arg_{L}\max\left(\sum_{i=1}^{n}\alpha_{i}-\frac{1}{2}\sum_{i,j=1}^{n}\alpha_{i}\alpha_{j}y_{i}y_{j}x_{i}x_{j}\right)$$

where $\alpha_{i}$ refers to the Lagrange multipliers, and the constraints are $\alpha_{i}\geq 0$ and $\sum_{i=1}^{n}\alpha_{i}y_{i}=0$.

On the other hand, SVM can be transformed into a nonlinear predictor by mapping the features into a higher dimension space. This new space can be approximated by replacing the x in Equation (18) by the kernel function $K\left(x_{i},x_{j}\right)$:

(19) $$L=\arg_{L}\max\left(\sum_{i=1}^{n}\alpha_{i}-\frac{1}{2}\sum_{i,j=1}^{n}\alpha_{i}\alpha_{j}y_{i}y_{j}K\left(x_{i},x_{j}\right)\right)$$

In this study, the Radial Basis Function (RBF) was used as the kernel function. The RBF can be written as follows:

(20) $$K\left(x_{i},x_{j}\right)=\exp\left(-\gamma {\left\|x_{i}-x_{j}\right\|}^{2}\right)$$

Support Vector Regression (SVR), which was developed by Vapnik et al. [66], is a revised version of SVM to apply for the regression problem. The difference from SVM is that SVR solves Equation (17) to find an f(x) having at the most ε deviation from the target value $y_{i}$. More detail of this regularization problem can be found in Awad and Khanna [67].

The Ridge regression model is a regularized linear regression. This model reduces the overfitting results by adding the regularization term into the weight coefficient. Since the overfitting increases the weight coefficient, Ridge regression can obtain a more accurate weight coefficient that indicates feature importance. In Ridge regression, the regularization is performed by minimizing the squared sum of weights with the squared sum of errors:

(21) $$w=\arg_{w}\min\left(\sum_{i=1}^{n}e_{i}{}^{2}-\lambda \sum_{j=1}^{m}w_{j}{}^{2}\right)$$

where w is the weight coefficient, e is an error, and λ is the scale parameter of regularization.

### 2.4. Feature Importance and Feature Selection

In this study, the six ML models mentioned above were divided into two groups by the feature importance metrics: mean decrease impurity and weight coefficient. First, the mean decrease impurity was used in the DT-based models (DT, RF, XGB). In the single DT model, the amount of performance improvement in each split node was calculated by the mean decrease of the node Gini index (Equation (5)) classification. The regression performance was obtained from the mean residual sum of squares. In ensemble DT models, the feature importance of all DTs within the model were averaged. A detailed theoretical background can be found in [68]. Second, indicating the feature importance of SVM and Ridge, the square of the weight coefficient in Equation (12) is the distance of each variable margin in the classification model. In terms of the classification, this means that the bigger the margin, the more precisely the significant variable is classified. From the aspect of regression, a weight coefficient $w_{i}$ of variable *i* quantifies the effect on the prediction ${\hat{y}}_{i}$, which indicates the feature importance of the regression predictor.

In the suggested BTC features, not all features are relevant to predict the spill location and spill mass. The redundant features may increase the modeling complexity, as well as leading to a decrease in the accuracy of ML models [69]. Moreover, excluding the redundant features is necessary to clarify the relationship between the BTC features and the contaminant source. Note that the information of the BTC implies the hydraulic and geometry characteristic of the transported reach in the river, which dominates the mixing characteristics of contaminants. Therefore, to predict the contaminant source, we can expand the significant BTC features to the dominant hydraulic and geometry factor.

In this study, recursive feature elimination cross-validation (RFECV) was employed to select the optimal feature sets of each model. RFE is a greedy algorithm to rank the features using the particular feature importance criteria of each model. This algorithm starts with a full set of features; it then removes the redundant feature repeatedly, until the model performance becomes poor. Then, the remaining features are selected as an optimal feature set. In addition, RFECV improves RFE with N-fold cross-validation, which can reduce the bias of the selected optimal feature set. As feature importance in RFECV, we utilized the feature importance criteria of each model for training each model by each selected feature set. RFECV was implemented using Scikit learn library in Python 3.7.

### 2.5. Modeling Performance Criteria

Due to the different tasks of approximating a mapping function, classifier and regressor were judged by different types of criteria. With respect to classifiers, accuracy, specificity, and sensitivity were used to measure the modeling performance, as shown in Equations (22)–(24):

(22) $$Accuracy=\frac{TP+TN}{TP+TN+FP+TN}$$

(23) $$Specificity=\frac{TP}{TP+FP}$$

(24) $$Sensitivity=\frac{TP}{TP+TN}$$

The number of true negatives (TN), false negatives (FN), true positives (TP), and false positives (FP) were used as the main components of the suggested criteria. The accuracy, specificity, and sensitivity show the overall ratio of accurate, negative, and positive prediction, respectively [27,70].

In the case of the regressor, *R*^2^ (coefficient of determination), Root Mean Square Error (MSE), Mean Absolute Error (MAE), and Mean Absolute Percentage Error (MAPE) were utilized to measure the quantitative error. The formulae are listed in Equations (25)–(28):

(25) $$R^{2}=1-\frac{\sum_{i}{\left(y_{i}-\hat{y_{i}}\right)}^{2}}{\sum_{i}{\left(y_{i}-\bar{y}\right)}^{2}}$$

(26) $$RMSE=\sqrt{\frac{1}{n}\sum_{i}{\left(y_{i}-\hat{y_{i}}\right)}^{2}}$$

(27) $$MAE=\frac{1}{n}\sum_{i}\left|y_{i}-\hat{y_{i}}\right|$$

(28) $$MAPE=\frac{1}{n}\sum_{i}\left|\frac{y_{i}-\hat{y_{i}}}{\hat{y_{i}}}\right|\times 100\%$$

where $y_{i}$ is the actual value and $\hat{y_{i}}$ is the prediction value. The RMSE is the square root of MAE, which has consistent units of target variables. The MAE is similar, which is calculated by the sum of the absolute error. The MAPE indicates a relative error, which is usually reported as a percentage. Regressors are ensured as better models when these criteria represent smaller values.

## 3. Study Site and Field Tracer Test

**Figure 4:**
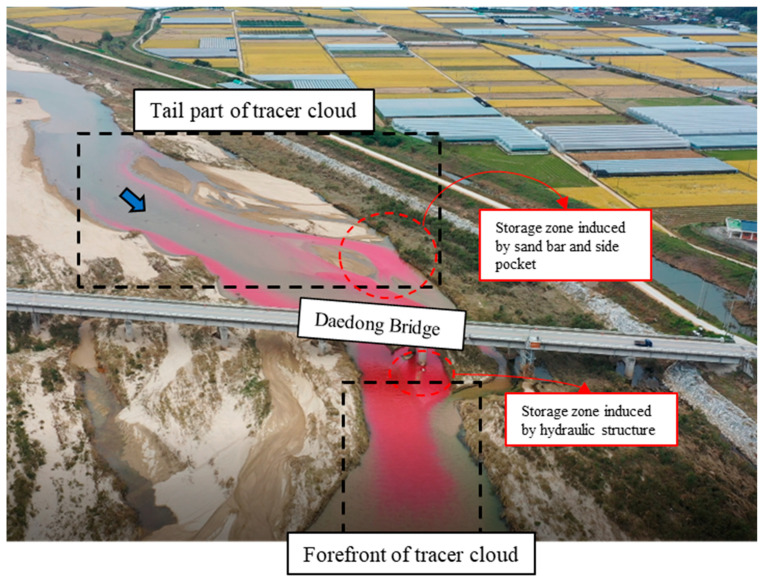
Photograph of the behavior of a tracer cloud that depicts the storage zone effect in the Gam Creek test reach.

The study site to apply the ITM framework in this study is the Gam Creek in Gimcheon City, South Korea. This river is located in the vicinity of an industrial complex, which poses a high risk of pollutant spill accidents. In addition, Figure 3 shows that it joins with the Nakdong River, where a large number of people and agriculture depend on the river as a water source. In terms of morphology, the Gam Creek is a typical braided river, of which the bed material is composed of sand substrate, and Figure 4 shows that the river contains plenty of storage zones, such as sand bars, vegetation, and side pockets.

The tracer tests used for field validation of the ITM framework were conducted under different streamflow conditions in October 2019 and June 2020. Figure 3 shows that the tests were conducted in the reach of Point 16 (injection point) to Point 20 (monitoring point). A fluorescent dye, Rhodamine WT, was used as a tracer material, which is a widely-used conservative tracer [43,71,72,73]. In Test 1 and Test 2, 15 and 7.5 L, respectively, of 20% Rhodamine WT solution were injected. Multiple point injection, according to the lateral direction of the channel, was conducted to achieve full mixing conditions in the horizontal and vertical direction for one-dimensional mixing conditions in the real stream. In addition, the distance between the injection point (IP) and Section 1 (S1) was estimated using Equation (29) [74]:

(29) $$L_{0}=0.1{\left(\frac{1}{n}\right)}^{2}\frac{UW^{2}}{E_{z}}$$

where $L_{0}$ is the distance from the injection point for complete mixing on cross-section, *n* is the number of injection points in the lateral direction, and $E_{z}$ is the lateral mixing coefficient, which is estimated from $E_{z}=0.15hU_{*}$ [40].

**Figure 5:**
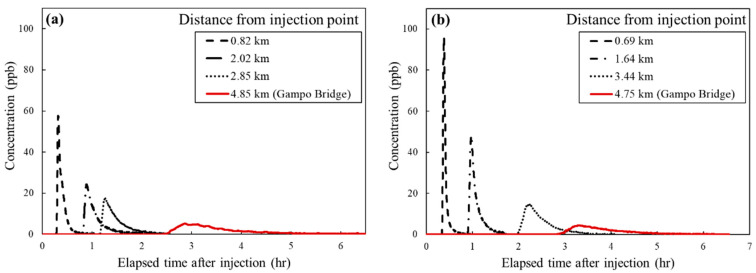
BTCs of (**a**) Test 1 and (**b**) Test 2 at different distances downstream of the injection point.

The Rhodamine WT was measured using YSI-600OMS fluorometry, and the concentration was calibrated using known concentration solutions in the range of 0 to 200 ppb. In order to obtain cross-sectional average concentrations, three or four sensors were installed laterally at uniform distance at all sites. Then, cross-sectional average concentrations were obtained by averaging the concentration data from all sensors in each section. Figure 4 is a photograph of Test 1 taken from a UAV, which was taken immediately after Rhodamine WT injection. In this figure, the anomalous spatial distribution was visualized with the storage zone effect from the sand bar, side pockets, and bridge piers. Due to these storage zone effects, the cross-sectionally averaged BTCs of Rhodamine WT showed a highly skewed and long-tailed shape. The discharge, velocity profiles, and water depth were measured using a Sontek Flowtracker acoustic Doppler velocimeter. The bottom slope was measured using a Sokkia GRX1 as Real-Time Kinematic-Global Positioning System (RTK-GPS). Table 3 shows the summarized hydraulic and geometry conditions of the field tracer tests. In Test 1, the discharge (*Q*) was six times larger than Test 2, so the mean width (*W*) and mean velocity (*U*) in Test 1 were greater than in Test 2. The tracer mass *(M)* was injected at twice the amount of Test 2 in Test 1. Figure 5 shows the BTCs of Test 1 and Test 2 at different distances downstream of the injection point. Although more tracers were injected in Test 1, the peak concentration was higher in Test 2 since the mass was diluted a lot due to the high discharge. Furthermore, the advection was more dominant, and dispersion was less than in Test 2, due to the high mean velocity.

## 4. Development of the ITM Framework in Gam Creek

### 4.1. Chemical Accident Scenarios in Gam Creek

In order to generate a training dataset for the proposed framework, breakthrough curves for the chemical accident scenarios in Gam Creek were created using CAS with TSM. Figure 3c shows that the spill scenarios were developed at 30 potential spill locations along the Gam Creek. The Hwangsan Bridge and Gampo Bridge were used as monitoring points in the Gam Creek to build the two inverse-tracking models represented as Model 1 and Model 2, respectively. For various flow conditions, 450 streamflow scenarios were generated from the log-normally fitted distribution using 10 years of historical streamflow data from an observation station located at Daedong Bridge. The streamflow data from 1 January 2010 through 31 December 2019 was obtained from the GIS-based Water Resources Management Information System (WAMIS) in South Korea. Using these sampled streamflow scenarios as input variables, the HEC-RAS was simulated to calculate hydraulic and geometry variables (*U*, U*, A, *h*) for estimation of the TSM parameters by Equation (3). The river geometry data and the Manning’s *n* coefficient of each cross-section were collected from the Master plan reports of Gam Creek [75]. The constructed HEC-RAS geometry consisted of 180 cross-sections within 39 km reach length. Manning’s *n* coefficient ranged (0.024–0.033). The sinuosity (*Sn*), which is a constant value, regardless of flow condition, was also estimated by the HEC-RAS geometry.

With respect to contaminant transport simulation, the total number of chemical accident scenarios was 13,500, which represented 30 potential spill locations for 450 streamflow scenarios. The spill mass was given to each scenario simulation from a randomly sampled value in the range of 0 to 10 ton. The spilled contaminants were assumed to be a conservative constituent that did not decay. In order to prepare simulation of chemical accident scenarios with the TSM model, the total model domain needs to be divided into sub-reaches having the same TSM parameter set. In this study, 48 sub-reaches were constructed by dividing the reaches into sections considering the river flow and geometric conditions, such as velocity, water depth, width, sinuosity, bridge, and tributaries. To achieve this, the averaged hydraulic and geometry variables were calculated to estimate the TSM parameters of the sub-reaches using empirical equations for TSM parameters (Equation (3)). Table 4 gives the statistics of the estimated reach averaged hydraulic, and geometry variables that served as input variables of Equation (3). In addition, Table 5 gives the estimated TSM parameters of each sub reach according to the streamflow scenarios. Notably, reasonable range values were calculated when compared with TSM parameters reported in previous studies [38,39,46].

Moreover, the Froude number (Equation (30)) of all streamflow scenarios represents that only subcritical flows were generated. Among the sampled streamflow values, the flow condition was only close to the supercritical flow with a Froud number of 0.94 at the maximum value of 129.51 m^3^/s. With the recognition that supercritical flow occurs at flood season, future studies should consider the hydrodynamic simulation with unsteady flow with the precipitation. In terms of numerical stability, it is necessary for reliable results to estimate the numerical error of simulated chemical accident scenarios. Silavwe et al. [76] suggested that the Peclet number (Equation (31)) of the Crank-Nicolson method-based 1D ADE should be less than 2 to avoid numerical error. Additionally, Choi [77] performed a numerical error test with the same TSM model as this study. The numerical error test showed that when the Peclet number did not exceed 5, oscillation-free solutions were obtained. Based on these results, the generated chemical accident scenarios were numerically stable due to the Peclet number of simulated chemical accident scenarios being in the range (0.20 to 2.23), as shown in Table 5. In Table 5, Froude number and Peclet number are defined as:

(30) $$Fr=\frac{U_{F}}{\sqrt{gh}}$$

(31) $$Pe=\frac{U_{F}\cdot \Delta x}{K_{F}}$$

### 4.2. Model Development

For training datasets to build predictors for both spill location and spill mass, the BTCs at two monitoring points were extracted from chemical accident scenarios of Gam Creek. Then, from these BTCs, BTC features were extracted and labeled with their spill location and spill mass for supervised learning. The development of the suggested framework consists of two steps. First, RFECV was used to identify the optimal feature subset of the ML algorithms and develop predictors for spill location and spill mass. In this step, 80% of the BTC features dataset was used as a training dataset and 20% was used as a test dataset. Second, five-fold cross-validation was conducted on the dataset to compare the performance of each ML model by optimal feature subset selected by RFECV. In this study, using the Ridge, DT, RF, XGB, and SVM classifier, two inverse tracking models were built, depending upon the monitoring points: Model 1 (Gampo Bridge) and Model 2 (Hwangsan Bridge), as shown in Figure 3c. Field application of the trained ML models for spill location and spill mass using field tracer test data is described in Section 5.

#### 4.2.1. BTC Feature Importance for Inverse Tracking the Contaminant Source

**Figure 6:**
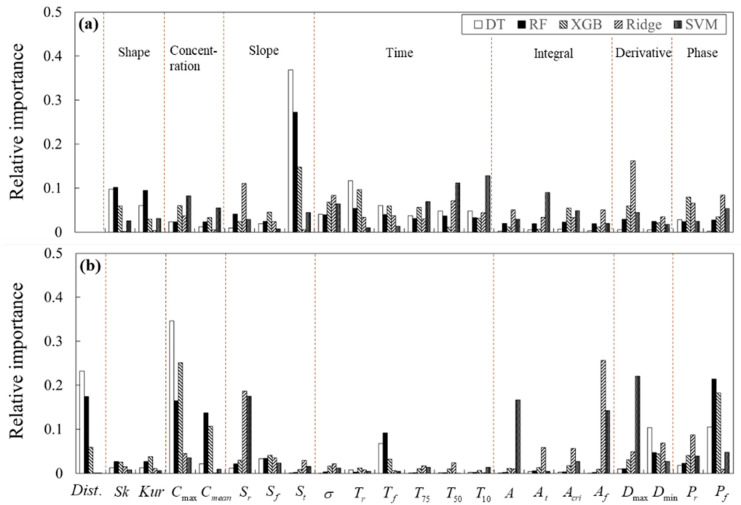
BTC feature importance by each ML model from Model 1 for (**a**) the prediction of the spill location and (**b**) the prediction of the spill mass.

In order to investigate the relevant BTC features for inverse tracking the spill location and spill mass of contaminant source, the importance of BTC features was estimated using the suggested ML models. All the feature importance was calculated to relative importance in the range 0 to 1. The feature importance of Model 1, which covers a more extended domain than Model 2, was plotted in Figure 6. In this figure, the first three bars are DT-based models using reduction of the Gini index as feature importance criteria, while the next two bars are Ridge and SVM using the weight coefficients as feature importance criteria. The feature importance values obtained by the reduction of the Gini index and the weight coefficients tended to be inversely proportional.

Figure 6a shows that for spill location prediction, the slope of the tail ($S_{t}$) proved to be the most crucial factor for the DT-based model. This feature represents the magnitude of the storage zone effect. The increase in the storage zone effect induces the long-tailed BTC, due to the trapping effect [78,79]. The duration above 50% and 10% of $C_{\max}$ ($T_{50}$, $T_{10}$) were relatively important for SVM. The $T_{50}$ depends on dispersion, while the $T_{10}$, which is the time length of the tail, is largely affected by the storage effect. The maximum derivative ($D_{\max}$) was relatively important for Ridge and XGB. This feature represents the derivative value when the concentration increases most rapidly in the rising limb of BTC. This feature is dominantly affected by the advection. However, compared to the DT-based model, the importance of the features was generally low in general in the SVM and Ridge. All of the feature importance of SVM and Ridge was under 0.2.

Figure 6b demonstrates that in spill mass prediction, the maximum concentration ($C_{\max}$) was the most important factor for the DT-based models. The distance and the falling limb area of the phase space ($P_{f}$) were also important features for the DT-based models. When the contaminant is spilled into the river, the $C_{\max}$ of the contaminant cloud decreases as it is transported downstream from the spill point. Thus, the distance and $C_{\max}$ can be judged as complementary factors to predict the spill mass. Additionally, the falling limb area of the phase space ($P_{f}$) represents the concentration reduction rate, which can be affected by the velocity and the storage zone. However, the slope of rising limb ($S_{r}$) and area of falling limb ($A_{f}$) were most important for SVM and Ridge regression models. Furthermore, SVR has more highly important features than Ridge regression, such as maximum derivative ($D_{\max}$), and total area (*A*). This can be explained by the different method of regularization of both models, as described previously. Since the Ridge regression regularizes the weight coefficient (Equation (16)), the feature importance can be underestimated. Consequently, the $S_{r}$ was the most important feature for spill mass prediction for SVM and Ridge regression models, and the $C_{\max}$ is the most important feature for spill mass prediction for tree-based models.

#### 4.2.2. Development of Spill Location Predictor

**Figure 7:**
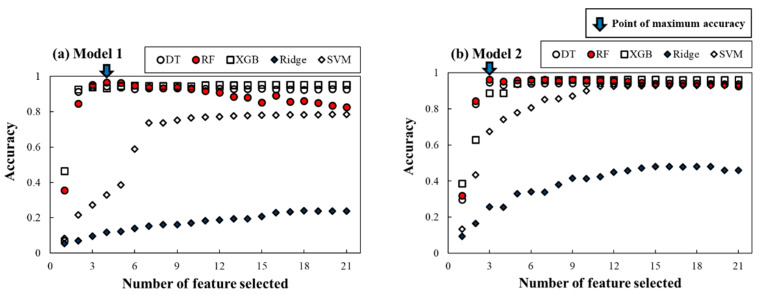
RFECV results of (**a**) Model 1 and (**b**) Model 2 for the spill location for finding the optimum number of features for each ML model.

In Figure 7a,b, RFECV with five-fold cross-validation was conducted based on accuracy as a score to identify the optimal feature subset. Table 6 represents the optimal hyperparameter set and selected optimal features. The best hyperparameter was investigated by grid search in the range based on previous study [80,81]. Parameters not listed followed the default settings of the Scikit-learn and Xgboost libraries [61,82].

The model performances were investigated through the three performance criteria described in the previous section. Table 7 represents the five-fold cross-validation results with all performance criteria as averaged values. From these results, DT ensemble models, RF and XGB, outperformed in all performance criteria: accuracy, sensitivity, and specificity all scored around 0.97, respectively. Meanwhile, Ridge and SVM-linear showed weak performance and produced a low-performance score. Moreover, the RF model not only showed the best performance, with an accuracy of 0.97, but also used only three and four variables as optimal features for Models 1 and 2, respectively. However, as the number of selected features grew, it showed overfitting. The results of SVM-RBF with a feature subset selected from SVM-linear showed that its performance was almost the same as the DT-based model, which is a significant improvement over the SVM-linear model. For most ML models, Model 2 showed better performance than Model 2, which means that the shorter the length of the model domain, the better the model performance.

#### 4.2.3. Development of Spill Mass Predictor

**Figure 8:**
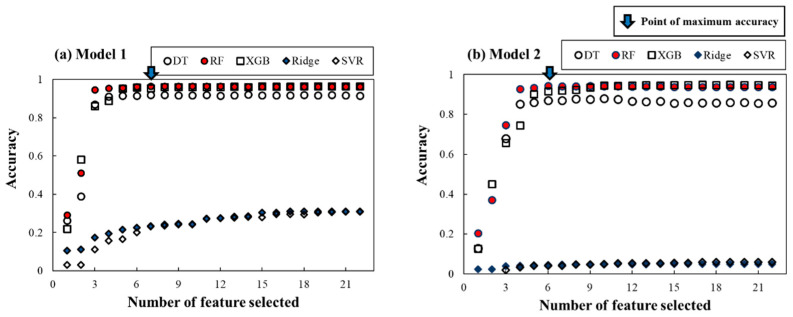
RFECV results of (**a**) Model 1 and (**b**) Model 2 for the spill mass for finding the optimum number of features for each ML model.

Spill mass models for Model 1 and Model 2 were also built by the Ridge, DT, RF, XGB, and SVR regression models, according to the monitoring points shown in Figure 2. Similar to the evaluation processes in the spill location models, RFECV and five-fold cross-validation were applied to find the optimal feature subset and, thus, optimal ML models. The results showed that among the ML models, RF shows the best accuracy of 0.97 (*R*^2^). RF also selected the smallest number of features: seven and six features in Models 1 and 2, respectively, as shown in Table 8. Unlike the spill location predictors, RF and XGB showed similar performance without overfitting, according to the number of selected features. In addition, the DT-based models also outperformed linear models, as shown in Figure 8. Table 9 summarizes the results of regression performance from the averaged five-fold cross-validation results with the four performance criteria. This table shows that RF and XGB outperformed the other ML model performances.

## 5. Field Application of ITM

The developed ML models were validated using the field tracer data obtained at Gam Creek. Among the measured Rhodamine WT concentration curves shown in Figure 5, the curves measured at Gampo Bridge were used as BTCs of the monitoring point of Model 2. Since the two tracer tests performed with different spill mass condition, the arrival time of Test 1 is earlier than Test 2 due to the faster flow condition, and the maximum concentration of Test 1 is lower than Test 2, because the flow rate of Test 1 was approximately five times that of Test 2. Compared with the synthetic BTC, the real BTC contained fluctuations due to channel irregularities and measurement error, as shown in Figure 5. Thus, this can cause a discrepancy with the BTC features of synthetic BTC, the validation of ML models with a field test is necessary.

### 5.1. Field Test of Spill Location Predictors

**Figure 9:**
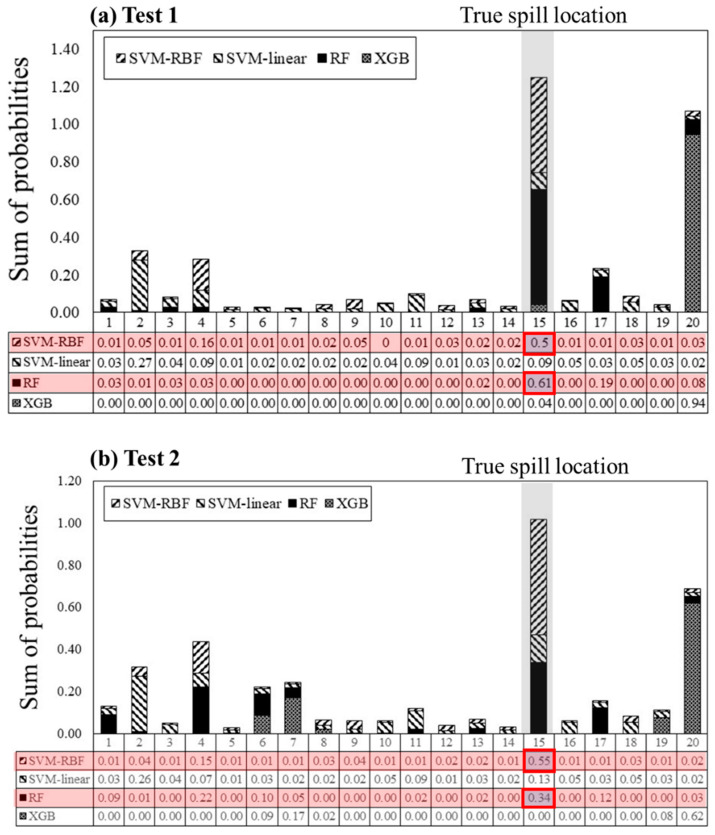
Predicted probability of spill location by using ML models using the measured BTC at the monitoring point (Gampo Bridge); The red boxes show the two highest values.

Figure 9 presents the prediction probability of ML models according to the potential spill locations. In this figure, we compared the ensemble DT-based models, RF and XGB, and SVM. In order to estimate the prediction probability, ensemble DT-based models estimate the mean predicted probabilities of the trees. The location probability of a single DT is the fraction of samples of the same location in a leaf. In SVM, the prediction probability was estimated by using Platt scaling, which fits the SVM output into probabilities by using an additional sigmoid function [83]. Both processes were achieved using the predict_proba (X) function in the Scikit-learn, which is a Python-based machine learning library.

The results show that only the SVM-RBF and RF predicted the correct spill location, showing Point 15 with the highest probability. In the case of Test 1, RF predicted the true spill location with 61% of probability, indicating a higher probability than SVM-RBF of 50%. In the case of Test 2, the SVM-RBF predicted the true spill location as a probability of 55%. This is higher than RF, which had a probability of 34%. This result was obtained because the slope of the tail and the time features, which are a value for time without a concentration value, was important for the prediction of the spill location.

On the other hand, the SVM-linear predicted the wrong location, and showed low probabilities for all locations. It can be seen that the linear model yields underfitting results, because the spill location and BTC features have a non-linear relation. However, the XGB, which showed similar accuracy to RF when validated with synthetic BTC, was rather poor in predicting the spill location. The results of XGB showed that in both cases, point 20, the closest location to the monitoring point (Gampo Bridge), was predicted as the spill location with 94 and 62% probabilities, respectively. This result implied that the trained model was overfitting. Additionally, it can be seen that RF is less sensitive to data noise than XGB because RF largely depends on time and slope features ($S_{t}$, $T_{r}$) that are less affected by noise. In conclusion, the XGB built a model that was too fit for the scenario-based training data set, and was not suitable in handling the field data. Hence, the parallel bagging method is more suitable to the application with field data, including more noise than the sequential boosting method in DT-based models. The noise is decreased in the bagging method by aggregating the single DT predictors in parallel.

In summary, both SVM-RBF and RF possess stable predictions, even with real concentration curves from field tests. However, it can be concluded that RF is not only the most accurate, but also the most efficient, with the smallest number of BTC features, namely, 3–4, as compared to SVM by utilizing all BTC features.

### 5.2. Field Test of Spill Mass Predictors

The spill mass predictors were also validated with the BTCs of the field tracer tests. The true spill mass values of Tests 1 and 2 were 3.48 and 1.74 kg, respectively. Table 10 demonstrates the true spill mass and estimated mass from RF, XGB, SVR-linear, and SVR-RBF. Additionally, the percent errors were used for comparison between ML models, as listed in Table 10. The prediction results show that for both tests, the XGB produced the smallest errors, while the estimations of the other models were found to involve high errors. Specifically, the SVR-linear diverged during the prediction. SVR-RBF showed better prediction results than the linear model, but both tests showed high errors. This means that the linear model is incapable of prediction with noisy data. Additionally, the SVR-RBF, which is well fitted with the BTCs of the scenarios, has no margin to be applied with the noisy data. In the case of RF, this model highly underestimated the spill mass close to 0 kg. From this result, it is evident that RF has low noise immunity, since the number of optimal features is small. In other words, RF is the same advanced DT model as XGB, but this model depends on only eight features, as described in Table 8. Thus, the high dependency on small features causes low noise immunity.

Consequently, XGB is the most feasible ML model for the prediction of spill mass in the field. In contrast to the spill location prediction, the boosting method of XGB showed a better result than the bagging method of RF. Additionally, the results show that the larger the number of optimal features, the better for spill mass prediction to apply in the field.

## 6. Conclusions

In this study, a practical framework of the Inverse Tracking Model (ITM) was developed to predict the spill location and mass of contaminants accidentally released into the river. In this framework, the numerical model of TSM was used to simulate the realistic BTCs of contaminant spill scenarios via reflecting a wide spectrum of river flow and mixing processes. From the contaminant spill scenarios, 21 features were extracted from the BTCs of a monitoring point, which indicate various characteristics of BTC. To build the optimal ML models for spill location and mass, we applied six ML models, and selected optimal BTC features using RFECV. The application and validation of the proposed framework were performed in Gam Creek, South Korea. From the results, the key conclusions and suggestions are as described below.

In the development of spill location predictors, the ensemble DT-based model, RF and XGB, outperformed other ML models. Furthermore, RF was the most efficient model, with a minimum number of optimum features. Among features of BTC, the slope of the tail ($S_{t}$), which characterizes the storage zone effect, played a significant role in predicting the spill location. From this result, it is evident that the tail of BTC implies the characteristic of the reach where contaminant transported due to the storage zone distributed in the reach. The SVM-RBF showed less accurate results than DT-based models in scenario-based validation results. In the development of spill mass predictors, RF and XGB showed better performance than the other ML models.

In the field application, for the prediction of spill location, the SVM-RBF was less affected by data noise of measured BTC from tracer tests than DT-based models due to the uniformly distributed BTC importance in field application. Nevertheless, from the aspect of the number of optimal features, RF was considered to be the most accurate and economical for the spill location prediction. For the prediction of the spill location, the XGB showed better field applicability than RF. In other words, the boosting method was more appropriate than the bagging method in the prediction of spill mass. Moreover, it could achieve more noise-immune models when using all BTC features.

The proposed framework has an advantage in that only the observed BTC is needed to predict the contaminant source characteristics, with no requirements of hydraulic or geometry information. However, it also has the limitation that the range of potential spill mass values to build the model is uncertain.

For future studies, some potential to improve the framework exists. First, the pulse injection should be taken into account for more various contaminant spill cases. Second, the unsteady flow with rainfall–runoff needs to be added into the contaminant spill scenarios. These improvements can be accomplished by minor modifications. Despite some remaining work for future study, the proposed framework will provide a practical and rigorous model for real-time application as a river accident response system.

## Figures and Tables

**Table 1:** Derived power of TSM empirical equations by PCR.

Parameter	*a*	*b*	*c*	*d*
*K_F_*	0.1955	1.3072	0.6631	1.0837
*A_F_*	−0.7098	0.1365	0.1213	0.0132
*A_S_*	−2.2661	−0.6268	0.3284	−1.4327
α	−4.8611	−0.4683	−0.5223	−2.1773

**Table 2:** Symbols and descriptions of BTC features.

BTC Features	Symbol	Description
Shape	Sk	Skewness
	K	Kurtosis
Concentration	$C_{\max}$	Maximum concentration
	$C_{mean}$	Mean concentration
Slope	$S_{r}$	The slope of rising limb
	$S_{f}$	The slope of a falling limb
	$S_{t}$	The slope of the tail by power-law regression
Time	σ	Standard deviation
	$T_{r}$	Duration of rising limb
	$T_{f}$	Duration of a falling limb
	$T_{75}$	Duration above 75% of $C_{\max}$
	$T_{50}$	Duration above 50% of $C_{\max}$
	$T_{10}$	Duration above 10% of $C_{\max}$
Integral	A	Total area
	$A_{t}$	Tail area
	$A_{cri}$	Critical area
	$A_{f}$	Falling limb area
Derivative	$D_{\max}$	Maximum derivative
	$D_{\min}$	Minimum derivative
Phase	$P_{r}$	Rising limb area of the phase space
	$P_{f}$	Falling limb area of the phase space

**Table 3:** Experimental condition of the field tracer tests in Gam Creek, South Korea.

	Date	Discharge (*Q*) [m^3^·s^−1^]	Reach Length (*L*) [km]	Mean Depth (*H*) [m]	Mean Width (*W*) [m]	Mean Velocity (*U*) [m/s]	Tracer Mass (*M*) [kg]
Test 1	17 October 2019	12.47	4.85	0.41	52.12	0.58	3.48
Test 2	4 June 2020	2.17	4.80	0.35	18.75	0.33	1.74

**Table 4:** Statistics of the estimated hydraulic and geometry variables of 48 sub reaches from HEC-RAS model simulations according to the streamflow scenarios in Gam Creek.

	*Q* (m^3^/s)	*S_0_*	*U* (m^3^/s)	U* (m^3^/s)	*A* (m^3^/s)	*W* (m)	*h* (m)	*Sn*
Mean	5.33	0.00571	0.34	0.051	13.17	83.46	0.17	1.0245
Std	10.83	0.00489	0.19	0.022	13.06	27.68	0.14	0.0590
Min	0.25	0.00003	0.04	0.006	0.57	46.38	0.02	1.0000
Max	129.51	0.04213	1.91	0.207	174.60	258.95	1.03	1.2687

**Table 5:** Statistics of the estimated TSM parameters of 48 sub reaches and the estimated non-dimensional parameters of the CAS scenarios in Gam Creek.

	*K_F_* (m^2^/s)	*A_F_* (m^2^)	*A_S_* (m^2^)	α (1/s)	*Fr*	*Pe* (dx = 15)
Mean	8.07	18.40	3.09	4.33 × 10^−5^	0.35	0.86
Std	7.91	17.04	2.17	1.91 × 10^−5^	0.12	0.36
Min	0.59	2.34	0.61	5.74 × 10^−6^	0.04	0.20
Max	91.80	238.03	31.35	1.44 × 10^−4^	0.92	2.23

**Table 6:** Hyperparameter and optimal feature subset from RFECV of the spill location predictor.

Method	Hyperparameter	Optimal Feature Subset (Number of Selected Features)
DT	-	Sk, $T_{r}$, $T_{f}$, $S_{t}$ (4)
RF	Num of tree = 100	Sk, K, $S_{t}$, $T_{r}$ (4)
XGB	Max_depth = 6, Min_child_weight = 1, Eta = 3, Subsample = 1, Colsample_bytree = 1	Sk, K, $C_{\max}$, $C_{mean}$, $S_{r}$, $S_{f}$, $S_{t}$, σ, $T_{r}$, $T_{f}$, $T_{50}$, $T_{10}$, $D_{\max}$, $D_{\min}$, $P_{r}$, $P_{f}$ (16)
Ridge	Alpha = 0.5	Sk, K, $C_{\max}$, $C_{mean}$, $S_{r}$, $S_{f}$, σ, $T_{r}$, $T_{f}$, $T_{75}$, $T_{50}$, $T_{10}$, $A_{t}$, $A_{cri}$, $D_{\max}$, $D_{\min}$, $P_{r}$, $P_{f}$ (18)
SVM-linear	C = 500, gamma = 1	Sk, K, $C_{\max}$, $C_{mean}$, $S_{r}$, $S_{f}$, $S_{t}$, σ, $T_{r}$, $T_{f}$, $T_{75}$, $T_{50}$, $T_{10}$, A, $A_{t}$, $A_{cri}$, $A_{f}$, $D_{\max}$, $D_{\min}$, $P_{r}$, $P_{f}$ (all 21 features)
SVM-RBF	C = 500, gamma = 1	Sk, K, $C_{\max}$, $C_{mean}$, $S_{r}$, $S_{f}$, $S_{t}$, σ, $T_{r}$, $T_{f}$, $T_{75}$, $T_{50}$, $T_{10}$, A, $A_{t}$, $A_{cri}$, $A_{f}$, $D_{\max}$, $D_{\min}$, $P_{r}$, $P_{f}$ (all 21 features)

Abbreviations: DT is Decision Tree; RF is Random Forest; XGB is Xgboost; SVM is Support Vector Machine; RBF is Radial Basis Function.

**Table 7:** Validation results of the spill location prediction models.

	Model 1			Model 2		
Method	Accuracy	Sensitivity	Specificity	Accuracy	Sensitivity	Specificity
DT	0.955	0.955	0.955	0.949	0.948	0.949
RF	0.968	0.968	0.969	0.975	0.974	0.975
XGB	0.952	0.952	0.952	0.966	0.966	0.967
Ridge	0.254	0.213	0.26	0.521	0.561	0.52
SVM-linear	0.868	0.868	0.868	0.974	0.974	0.974
SVM-RBF	0.943	0.944	0.943	0.975	0.975	0.975

**Table 8:** Hyperparameter and optimal feature subset from RFECV of the spill mass predictor.

Method	Hyperparameter	Optimal Feature Subset (Number of Selected Features)
DT	-	Sk, K, $C_{\max}$, $C_{mean}$, $S_{r}$, $S_{f}$, $S_{t}$, $T_{f}$, A, $A_{t}$, $D_{\max}$, $D_{\min}$, $P_{r}$, $P_{f}$ (14)
RF	Num of tree = 100	*Distance*, K, $C_{\max}$, $C_{mean}$, $S_{f}$, $S_{t}$, $D_{\min}$, $P_{f}$ (8)
XGB	Max_depth = 7, Min_child_weight = 3, Eta = 0.3, Subsample = 0.5, Colsample_bytree = 0.7	*Distance*, Sk, K, $C_{\max}$, $C_{mean}$, $S_{r}$, $S_{f}$, $S_{t}$, σ, $T_{r}$, $T_{f}$, $T_{75}$, $T_{50}$, $T_{10}$, A, $A_{t}$, $A_{cri}$, $D_{\max}$, $D_{\min}$, $P_{r}$, $P_{f}$ (all 21 features)
Ridge	Alpha = 0.5	Sk, K, $C_{\max}$, $C_{mean}$, $S_{r}$, $S_{f}$, $S_{t}$, σ, $T_{r}$, $T_{f}$, $T_{75}$, $T_{50}$, $T_{10}$, A, $A_{t}$, $A_{cri}$, $A_{f}$, $D_{\max}$, $D_{\min}$, $P_{r}$, $P_{f}$ (all 21 features)
SVR-linear	C = 100, gamma = 10	Sk, K, $C_{\max}$, $C_{mean}$, $S_{r}$, $S_{f}$, $S_{t}$, σ, $T_{r}$, $T_{f}$, $T_{75}$, $T_{50}$, $T_{10}$, A, $A_{t}$, $A_{cri}$, $A_{f}$, $D_{\max}$, $D_{\min}$, $P_{r}$, $P_{f}$ (all 21 features)
SVR-RBF	C = 100, gamma = 1	Sk, K, $C_{\max}$, $C_{mean}$, $S_{r}$, $S_{f}$, $S_{t}$, σ, $T_{r}$, $T_{f}$, $T_{75}$, $T_{50}$, $T_{10}$, A, $A_{t}$, $A_{cri}$, $A_{f}$, $D_{\max}$, $D_{\min}$, $P_{r}$, $P_{f}$ (all 21 features)

**Table 9:** Validation results of the spill mass prediction models

Method	Model 1				Model 2			
	R^2^	MAE	RMSE	MAPE	R^2^	MAE	RMSE	MAPE
DT	0.937	0.538	0.734	15.808	0.888	0.919	0.959	18.731
RF	0.971	0.246	0.496	14.544	0.960	0.325	0.570	15.745
XGB	0.972	0.242	0.492	20.458	0.960	0.325	0.570	16.634
Ridge	0.341	5.636	2.374	279.57	0.228	6.316	2.513	185.72
SVR-linear	0.272	6.220	2.494	190.73	0.221	6.3742	2.5247	171.83
SVR-RBF	0.894	0.906	0.952	45.043	0.887	0.923	0.961	38.450

Abbreviations: RMSE is Root Mean Square Error MSE is Mean Square Error; MAE is Mean Absolute Error; MAPE is Mean Absolute Percentage Error.

**Table 10:** Predicted spill mass of ML models using the measured BTCs of Tests 1 and 2.

Method	Test 1			Test 2		
	*M* (kg)	*M_est_* (kg)	*ΔM* (%)	*M* (kg)	*M_est_* (kg)	*ΔM* (%)
RF	3.48	0.004	99	1.74	0.003	99
XGB	3.48	2.62	25	1.74	1.73	0.6
SVR-linear	3.48	-	-	1.74	-	-
SVR-RBF	3.48	5.40	−55	1.74	5.39	−210
